# Periodontal Manifestation in a Patient with Kindler Syndrome

**DOI:** 10.1155/2021/6671229

**Published:** 2021-03-08

**Authors:** Aysegul Sari, Salih Celik

**Affiliations:** ^1^Faculty of Dentistry, Department of Periodontology, Hatay Mustafa Kemal University, Hatay, Turkey; ^2^Department of Oral and Maxillofacial Surgery, TDC Dental Clinic, Antalya, Turkey

## Abstract

Kindler syndrome is a rare subtype of inherited epidermolysis bullosa. A 42-year-old female patient was admitted to our clinic with a complaint of tooth mobility. Multiple hypo- and hyperpigmented macules dissipated all over her body, prominent poikilodermatous changes, xerosis of the skin, and atrophy were seen in the clinical extraoral examination. Intraoral examination showed atrophy of the buccal mucosa, limited oral opening, epidermal tissue easily separated from the connective tissue, painful ulcers of the hard palate, severe periodontitis, and keratosis of the lips. All of the teeth showed mobility. After dermatologist consultation, the diagnosis of the patient was clinically identified as “Kindler syndrome.” All of her teeth were extracted due to her progressive periodontal disease and late admission to our clinic. Periodontal treatment might be effective in treating and controlling oral symptoms related to the syndrome and in improving the patient's quality of life.

## 1. Introduction

Kindler syndrome (KS) is defined as a rare autosomal recessive genodermatosis disease. Progressive poikiloderma, the presence of trauma-induced blisters, diffuse cutaneous atrophy, abnormal pigmentation, varying degrees of photosensitivity, and skin fragility are prominent clinical characteristics of the syndrome. Theresa Kindler described the syndrome for the first time in a 14-year-old girl with acral blistering since childhood who subsequently developed photosensitivity and poikiloderma in 1954 [1].

The genetic origin of the syndrome was first defined in 2003, with the identification of loss-of-function mutations in the gene KIND1 held on chromosome 20p12.3 [2]. More than 25 mutations have been revealed in this gene. Gene KIDIN 1 encodes kindlin-1 protein, which is one of the components of focal contacts in keratinocytes expressed particularly in the basal keratinocytes located in the epidermis. Abnormal skin fragility with defects in the actinextracellular matrix linkage is caused by loss of this protein [3, 4].

Histopathological examination of the cutaneous biopsy in KS reported a presence of pigmentary incontinence, degeneration of focal vacuole in the basal layer accompanied by subepidermal cleft, dilatation of blood vessels in the upper dermis, and epidermal atrophy [5, 6].

Other clinical symptoms of KS include nail dystrophy; acral hyperkeratosis [7]; webbing and contractures of toes and fingers [6]; alopecia [8]; actinic changes [9]; mucosal involvement including esophageal [6], oral commissure [10], vaginal [6], and urethral [8] stenosis; ectropion of the eyelids [10]; pigmentation of the lips [8]; and onychodystrophy [6].

The present case reported periodontal management of a 42-year-old female patient KS.

## 2. Case Report

**Figure 1:**
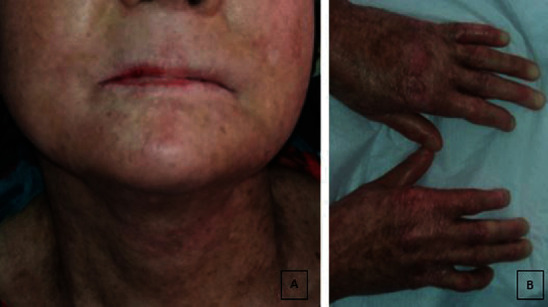
(a) The image showing multiple hypo- and hyperpigmented macules, prominent poikilodermatous changes, xerosis, alopecia, and atrophy of the skin. (b) The image showing cigarette paper-like wrinkling on the dorsum of the feet and hands. Atrophy and adhesions were detected in the fingers.

A 42-year-old female patient presented to Hatay Mustafa Kemal University, Faculty of Dentistry, Periodontology Department, with complaints of tooth mobility in February 2016. Multiple hypo- and hyperpigmented macules dissipated all over her body, prominent poikilodermatous changes, xerosis of the skin, alopecia, and atrophy were seen in the clinical extraoral examination. There was a distinct cigarette paper-like wrinkling on the dorsum of the feet and hands. Atrophy and adhesions were present in the fingers (Figure 1). Her mental and motor statuses were normal.

**Figure 2:**
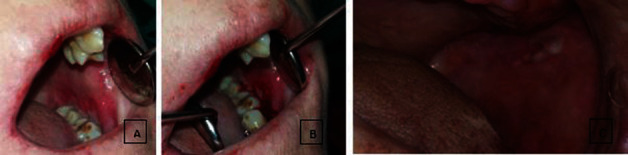
(a) The image showing atrophy of the lip, painful ulcers of the buccal mucosa, and keratosis of the lips. (b) It was clinically detected that the oral epidermal tissue easily separated from the connective tissue. (c) Leukoplakia-like lesions were observed in the buccal mucosa.

**Figure 3:**
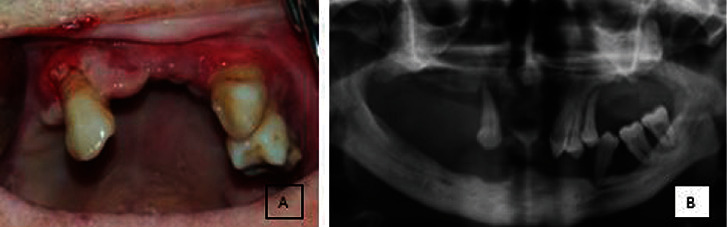
(a) The image showing that the clinical attachment loss was severe in the periodontal tissue. Bleeding, swelling, atrophy, and floppy were observed in the gingival tissue. (b) The panoramic radiography image of the patient.

Intraoral examination showed atrophy of the buccal mucosa, limited oral opening, epidermal tissue easily separated from the connective tissue, painful ulcers of the hard palate, severe periodontitis, and keratosis of the lips. Also, leukoplakia-like lesions were observed in the buccal mucosa (Figure 2). Clinical attachment loss was severe in the periodontal tissue. There were few teeth in the mouth. All of the teeth showed mobility. The gingiva was thin and fragile. Bleeding, swelling, atrophy, and floppy were observed in the gingival tissue (Figure 3).

**Figure 4:**
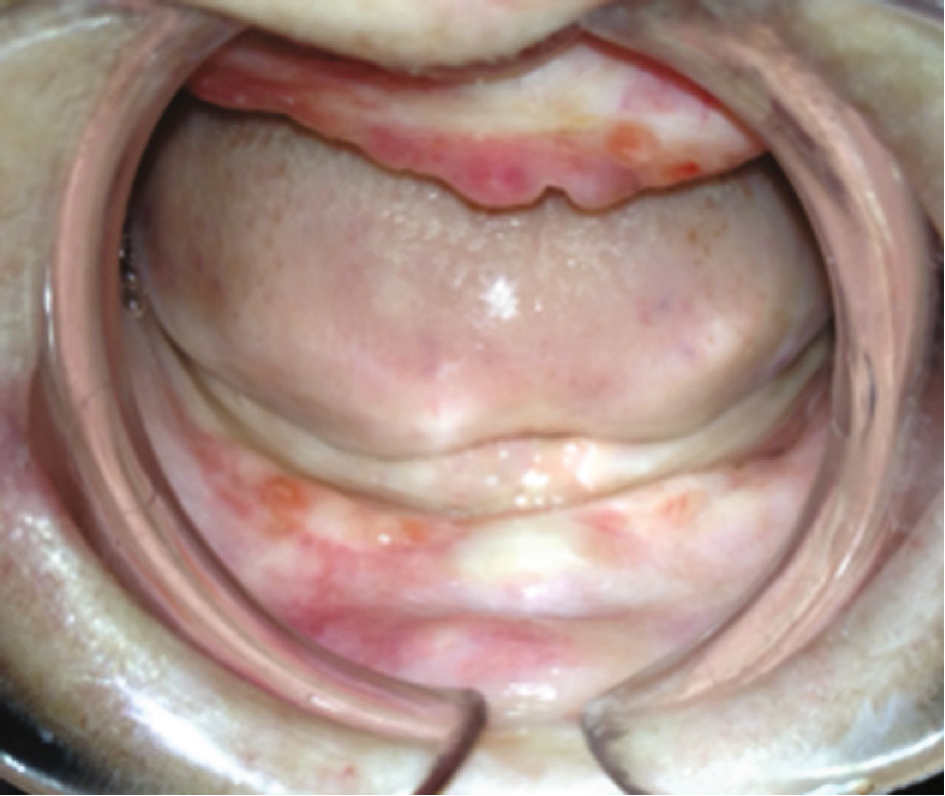
The image showing six-week postoperative healing after medicine treatment and gingivectomy.

The patient stated that she had a syndrome in her anamnesis; however, she would not give to us sufficient information about her disorder. Hospital records of the patient could not be reached. The patient was referred to the dermatology department for a definitive diagnosis. The patient's disorder was diagnosed as “Kindler syndrome” by the dermatologist. All of her teeth were extracted due to her progressive periodontal disease and late admission to our clinic. Furthermore, floppy gingival tissue was removed with gingivectomy for tissue modeling (Figure 4). She was prescribed 0.2% chlorhexidine gluconate for mouth rinsing (2x1, during 14 days), in addition to tetracaine chlorhydrate (0.5 mg) and hexamidine isethionate (1 mg) solution for relieving the oral symptoms.

Conservative treatment was preferred since the results of surgical and dental implant treatments to the patient were not predictable. The patient was referred to the prosthodontics department for the prosthetic process and maintained under clinical follow-up.

## 3. Discussion

Kindler syndrome is a rare heritable skin disorder with a complex phenotype and poorly understood pathogenesis [11, 12]. The current case reported the oral findings of a 42-year-old female patient with KS who applied to our clinic when her periodontal prognosis had advanced.

KS has various clinical symptoms such as acral skin blisters, progressive poikiloderma [1], alopecia, nail dystrophy, acral hyperkeratosis [7], contractures and webbing of toes and fingers [6], photosensitivity [1], and actinic changes [9]. In addition, oral symptoms are very common in this disorder. It was reported that oral manifestations often include severe periodontitis which begins with permanent teeth eruption and progresses rapidly, poor dentition with premature loss of teeth, erosive areas in the labial and buccal mucosa and gingiva, spontaneous bleeding, desquamative gingivitis, angular cheilitis, leukokeratosis of the lips, caries, halitosis, and xerostomia [13]. Clinical and oral manifestations of the present case were similar to those reported in previous studies.

Oral ulcerations and rapidly progressing periodontitis are conditions that should be considered in terms of periodontal health in patients with KS. Kindlin-1 mutations can cause these symptoms [14]. Kindler syndrome is a genetic disorder that occurs as a result of mutations in the fermitin family homolog 1 gene that encodes the kindlin-1 protein which plays a role in cell adhesion, spreading, and migration [15]. It has been reported that kindlin-1 has a basic role in actin-dependent keratinocyte cell adhesion, which is necessary for epidermal and periodontal health. Lack of this protein in keratinocytes results in reduction of cell spreading, proliferation, and migration rate [16]. Larjava et al. indicated that rapid progression of periodontal disease can be caused by deficiency of integrin activation in the junctional epithelium which can be caused by kindlin-1 mutations [17].

There are few studies evaluating periodontal condition in patients with KS. Wiebe et al. [18] reported that in a case report, by periodontal therapy and long-term follow-up, many teeth were maintained for >10 years despite the rapid prognosis of periodontal disease in patients with KS. Siegel et al. suggested that periodontitis progression was rapid in patients with KD compared to healthy controls [19]. Also, Wiab et al. indicated that kindler subjects exhibited largely similar patterns of periodontal destruction on both sides of the mouth [20]. In accordance with previous data, periodontal disease prognosis was very advanced in the present case. The patient's admission for treatment was in the late period, which limited the periodontal treatment. Since all the teeth had serious mobility and almost all periodontal attachments were lost, all of the teeth were extracted. It can be considered that periodontal disease process could have been accelerated because the patient had never received periodontal treatment before.

## 4. Conclusion

Management of patients with Kindler syndrome requires a multidisciplinary approach. Periodontal treatment might be effective in treating and controlling oral symptoms related to the syndrome and in improving the patient's quality of life.
